# Dietary assessment of British police force employees: a description of diet record coding procedures and cross-sectional evaluation of dietary energy intake reporting (The Airwave Health Monitoring Study)

**DOI:** 10.1136/bmjopen-2016-012927

**Published:** 2017-04-04

**Authors:** Rachel Gibson, Rebeca Eriksen, Kathryn Lamb, Yvonne McMeel, Anne-Claire Vergnaud, Jeanette Spear, Maria Aresu, Queenie Chan, Paul Elliott, Gary Frost

**Affiliations:** 1 Nutrition and Dietetic Research Group, Faculty of Medicine, Imperial College, London, UK; 2 Department of Epidemiology and Biostatistics, MRC-PHE Centre for Environment and Health, School of Public Health, Imperial College, London, UK

**Keywords:** NUTRITION & DIETETICS, OCCUPATIONAL & INDUSTRIAL MEDICINE, EPIDEMIOLOGY

## Abstract

**Objectives:**

Dietary intake is a key aspect of occupational health. To capture the characteristics of dietary behaviour that is affected by occupational environment that may affect disease risk, a collection of prospective multiday dietary records is required. The aims of this paper are to: (1) collect multiday dietary data in the Airwave Health Monitoring Study, (2) describe the dietary coding procedures applied and (3) investigate the plausibility of dietary reporting in this occupational cohort.

**Design:**

A dietary coding protocol for this large-scale study was developed to minimise coding error rate. Participants (n 4412) who completed 7-day food records were included for cross-sectional analyses. Energy intake (EI) misreporting was estimated using the Goldberg method. Multivariate logistic regression models were applied to determine participant characteristics associated with EI misreporting.

**Setting:**

British police force employees enrolled (2007–2012) into the Airwave Health Monitoring Study.

**Results:**

The mean code error rate per food diary was 3.7% (SD 3.2%). The strongest predictors of EI under-reporting were body mass index (BMI) and physical activity. Compared with participants with BMI<25 kg/m^2^, those with BMI>30 kg/m^2^ had increased odds of being classified as under-reporting EI (men OR 5.20 95% CI 3.92 to 6.89; women OR 2.66 95% CI 1.85 to 3.83). Men and women in the highest physical activity category compared with the lowest were also more likely to be classified as under-reporting (men OR 3.33 95% CI 2.46 to 4.50; women OR 4.34 95% CI 2.91 to 6.55).

**Conclusions:**

A reproducible dietary record coding procedure has been developed to minimise coding error in complex 7-day diet diaries. The prevalence of EI under-reporting is comparable with existing national UK cohorts and, in agreement with previous studies, classification of under-reporting was biased towards specific subgroups of participants.

**Strengths and limitations of this study**

- The Airwave Health Monitoring Study provides the largest collection and assessment of 7-day food records from a single occupation UK cohort.
- A comprehensive and reproducible diet record coding procedure has been developed to minimise coding error in this study.
- A large number of occupational as well as socio-demographic variable measures facilitate the investigation of a wide range of factors potentially associated with energy intake (EI) misreporting.
- Self-report physical activity and dietary data highlight the common limitations of estimating accuracy of dietary EI reporting in large nutritional epidemiological studies

## Introduction

The Airwave Health Monitoring Study is a longitudinal study of British police force employees launched in 2004.^1^ This study is the largest cohort of police employees worldwide with 42 112 participants enrolled into the study at the end of 2012 with a high proportion of men in early adulthood who are unrepresented in existing UK longitudinal studies.^2^ Data from the voluntary health-screening programme include extensive occupational, medical, biochemical, cognitive and lifestyle information. Since April 2007, participants completed a 7-day estimated weight food diary (*n* 15 404). One of the limitations of previous research in large-scale occupational cohort studies is that retrospective methods of dietary data collection, such as food frequency questionnaires,^3^ ^4^ rather than prospective methods have been used to investigate dietary behaviour. Therefore, the large-scale collection of 7-day food records from a single occupational group makes the Airwave Health Monitoring Study unique, as it will allow the comprehensive investigation of diet and various occupational factors with health outcomes.

The benefit of prospective measurement methods such as diet diaries compared with food frequency questionnaires is that they allow more detailed dietary intake to be captured, as they do not measure against a predefined food list. Additionally, compared with food records they are less reliant on participant recall. Prospective measures such as diet diaries provide important information about eating occasions, frequency of eating, regularity and the combination of foods consumed. Research increasingly suggests that measuring these additional aspects of dietary behaviour provides a holistic understanding about the relationship between diet and various chronic metabolic diseases.^5–8^ Despite the valuable information generated from diet diaries, it is widely acknowledged that all current dietary measurement tools present a challenge to nutritional scientists as they are subject to human error at each stage of the assessment process. First, there may be either intentional or unintentional misreporting of dietary intake by participants.^9^ For example, prospective dietary reporting may result in conscious or unconscious changes in diet intake during the period of observation. This is of particular concern, as energy balance is an established risk factor in the aetiology of chronic metabolic diseases. It is also acknowledged that dietary misreporting may be associated with specific population groups.^10^ ^11^ Therefore, an important part of the methodological process in nutritional epidemiology is to investigate the plausibility of dietary energy reporting and to identify participant characteristics associated with implausible reporting to avoid bias and erroneous conclusions.^12^ The second stage open to error is the ‘coding’ of food records (the matching of food and drink items recorded to a nutritional database code and a portion size) which is prone to subjective decision-making even by experienced coders.^13^

The recently published Strengthening the Reporting of Observational Studies in Epidemiology-Nutritional Epidemiology (STROBE-nut) statement recommends transparency in the methods used to derive nutritional data and the investigation of potential sources of bias in dietary reporting.^14^ In line with STROBE-nut recommendations, the aims of this paper are (1) to describe the dietary data coding methods applied to the Airwave Health Monitoring Study cohort, (2) to investigate the plausibility of energy reporting among the Airwave Health Monitoring Study participants and (3) to identify the characteristics associated with energy intake (EI) misreporting. We also conducted exploratory analyses to determine if diet code error rate was associated with implausible EIs. The results of this study will characterise dietary energy reporting within the Airwave Health Monitoring Study and will be used in subsequent studies to guide the statistical treatment of nutritional intakes within this cohort.

## Methods

The Airwave Health Monitoring Study is conducted according to the guidelines laid down in the Declaration of Helsinki. Written informed consent was obtained from all participants.

### Description of dietary record coding methods

#### Dietary measurement

The Airwave Health Monitoring Study was open to all police forces in Great Britain. Recruitment procedures have been previously described in detail.^1^ Dietary intake was measured using 7-day estimated weight food diaries previously validated against urinary and blood biomarkers in a large-scale UK epidemiological cohort.^15^ The food diary was posted to participants with detailed written instructions to record all food and drink consumed over seven consecutive days in predefined eating occasions. Participants were asked to provide details on cooking methods, brand names and portion sizes. To aid portion size estimation, photographs were provided based on those developed by Nelson and Haraldsdottir.^16^

#### Dietary data generation

Calculation of nutritional intake was conducted using Dietplan6.7 software (Forestfield Software, Horsham, UK) which is based on the McCance and Widdowson's 6th Edition Composition of Foods UK Nutritional Data set (UKN). A team of trained coders ‘coded’ the diaries (matching of food and drink items recorded to a UKN database code and a portion size). Diaries were excluded from coding when <1 day was completed or if a meal replacement diet was recorded. A standard operating protocol was developed to reduce the number of subjective decisions made by coders. It provides a series of flow diagrams to guide coders in the translation of food and drink records to database codes and portion sizes to weights (grams) using published recourses of portion^17^ ^18^ and food density information.^18^ ^19^ The standard operating protocol is available as a supplmentary document (see online supplementary document). In conjunction with the standard protocol, a ‘codebook’ has been developed to assist decision-making when no exact UKN code match can be found. The codebook is an evolving database containing >600 online supplementary codes and coding rules following the principles of the codebook designed by Conway *et al* for use in the International collaborative of macronutrients, micronutrients and blood pressure (INTERMAP) study.^20^ Examples of different scenarios and possible coding solutions are shown in online supplementary material table S1.

10.1136/bmjopen-2016-012927.supp1 supplementary document

10.1136/bmjopen-2016-012927.supp2 supplementary table

#### Coder training

To date, 20 dietary coders have been trained in the use of Dietplan6.7 software and the Airwave Health Monitoring Study standard protocol. The time an individual coder works on the project varies between 3 months to 2 years. All trainees are required to code ten ‘test’ food diaries using the standard protocol and codebook before progressing to code diaries from the study. A research dietitian or nutritionist checks the completed electronic Dietplan6.7 record against the written diary for coding errors. Individual feedback is given to the trainee coder after each test diary is completed. At this time, if the total errors are >10% per diary, the coder will be required to complete further test diaries until errors are within tolerance.

#### Quality checking

An audit cycle has been developed to monitor intercoder reliability with the aim of continuously improving coding consistency. Five per cent of all coded diaries are selected at random every 2 months. Research dietitians and nutritionists check the selected electronic Dietplan6.7 record against the written food diary and classify errors as: ‘code selection error’ (the code selected in Dietplan6.7 does not match the written record), ‘portion error’ (over ±10% difference of the protocol weight), ‘meal code error’ (item entered into incorrect meal occasion), ‘missing code error’ (item not coded, ie, in the written record) and ‘extra code error’ (item coded, ie, not in the written record). If the error rate in an audit check diary is >10%, feedback and training is provided to the individual coder. Following each audit cycle, the results are fed back to the team and coding improvement strategies are implemented as indicated, for example, staff training, new codebook entries and the development of additional protocol flow diagrams. The final data set is screened for gross coding errors. A gross coding error is defined as when the quantity of food recorded is a clear code error,^21^ for example, entering 260 g of instant coffee powder rather than 260 g of instant coffee made with water. If the quantity of any item coded exceeded the set maximum portion, the food diary barcode was identified and the original diary record checked and the quantity amended.

### Evaluating reported EI

#### Participants

Inclusion criteria for the present cross-sectional analysis were men and women enrolled into the Airwave Health Monitoring Study between 2007 and 2012 who had health screen and coded dietary data without gross coding errors (*n* 4412). Dietary energy under-reporting methods assume a stable bodyweight, therefore participants were excluded from the analyses if they were pregnant (*n* 0) and/or reported being on a weight-loss diet at the time of the health screen (*n* 317). Two participants were excluded due to extremely low EI reporting of <500 kcal/day, which is considered to be physiologically unsustainable.

#### Non-dietary variable measurements

During the health screen, participants were asked to complete a self-administered questionnaire on a touch screen computer to recorded information about occupational, lifestyle, medical history, socioeconomic and demographic factors. Total working hours were taken as the sum of ‘regular weekly working hours’ plus ‘usual overtime hours’ and classified into four groups (<41, 41–48, 49–54 and >55 hours per week) based on previous research.^22^ Standard police employment rank for officers was selected from a predetermined list (constable/sergeant, inspector/chief inspector and superintendent/higher). Participants not employed as police officers were classified as ‘staff’. Job descriptions were collapsed from 31 to two categories of ‘job role’ based on predominant working environment (mobile or office based). Trained nurses took anthropometric measurements following a standard protocol. Bodyweight was measured to the nearest 0.05 kg using digital scales (Marsden digital weighing scale). Standing height was measured to the nearest 0.1 cm (Marsden H226 portable stadiometer). Body mass index (BMI kg/m^2^) was classified as per WHO cut-offs: underweight <18.5 kg/m^2^; healthy 18.5–24.99 kg/m^2^; overweight 25–29.99 kg/m^2^ and obese >30 kg/m^2^.^23^ Physical activity information was collected using The International Physical Activity Questionnaire-Short Form (IPAQ-SF)^24^ which calculates metabolic equivalent (MET) minutes per week across three exercise parameters (walking, moderate and vigorous). The IPAQ-SF protocol was followed to classify each participant as achieving a high, moderate or low level of activity.^25^ As the physical activity data generated by IPAQ-SF do not cover a 24-hour period to permit translation to an overall physical activity level (PAL) value, we assigned estimated PALs of 1.4, 1.6 and 1.8 to ‘low’, ‘moderate’ and ‘high’ IPAQ-SF MET classification, respectively. These values are based on published Department of Health guidance representing non-occupational and occupational activity levels.^26^ To check the rationale of this assumption, we compared IPAQ-MET classification with self-reported body type. Self-reported body type was asked by the research nurse as part of the bioelectrical impedance measurement protocol (Tanita BC-418MA body composition analyser). ‘Athletic’ refers to intense exercise (>10 hours intense exercise per week) and ‘standard’ to <10 hours intense exercise per week. The agreement between those self-classified as ‘athletic’ and being in the highest IPAQ-SF MET category was 100%. To explore if coder error rate was related to EI misreporting, we classified coders dichotomously (mean error rates above/below the overall mean error rate of 3.7%).

#### Classification of EI misreporting

Potential misreporting of EI reporting is based on the assumption that participants are weight stable, where estimated EI is equal to estimated total energy expenditure (TEE). EI was calculated as the mean daily EI recorded across the number of days the food diary was completed. TEE is expressed as estimated basal metabolic rate (BMR) multiplied by an estimated PAL value. We estimated individual BMR (kcal/day) using Schofield equations based on sex, age and weight;^26^ we then assigned PAL values based on the MET category and applied the Goldberg equation.^27^ This equation takes into account the estimated variation in daily EI, BMR and PAL based on previous studies and the number of days of diet assessment.^27^ CIs were calculated for each participant based on PAL values and days of food diary completion. Participants with a ratio of EI:BMR below the lower 95% CI cut-off value were classified as possibly under-reporting and above the higher cut-off as potentially over-reporting EI. There were 15 (0.3%) participants classified as over-reporting EI; these were removed from subsequent analyses due to their small number, providing a final analytical sample size of 4078.

#### Statistical methods

Statistical analyses were undertaken using Statistical Analysis System’s statistical software V.9.3 (SAS Institute, Cary, North Carolina, USA). All statistical tests are two sided. The sample was stratified by sex to allow estimation of sex-specific associations between variables and the likelihood of implausible energy reporting. To assess differences between two groups, independent t-tests were used for data with normal distribution and Mann-Whitney U test otherwise. Mean and SD were reported for data with normal distribution and median and IQR otherwise. Associations with categorical variables were analysed using test (χ²). Sex-stratified stepwise logistic regression was conducted to identify variables that showed a statistically significant association with likelihood of under-reporting EI. Initially, we included all variables known to be previously associated with EI misreporting (age, BMI, smoking status, household income, physical activity, ethnicity, marital status and education) and occupational specific covariates related to the Airwave Health Monitoring Study (police rank, job role, number of working hours, time sitting per weekday). Shift work was not included in the model due to the small sample size with available data *(n* 536). Job role (*n* 2875) was not a significant predictor of under-reporting in the initial models, and due to its significant association with rank (χ², p=0.048) and weekday sitting (χ², p<0.0001), this variable was not included in the final model to enable the maximum sample size with complete data to be included for analysis. Variables with p<0.05 were included in the final model. Two sets of sensitivity analyses were conducted: one removing participants who reported being on a special diet not for weight loss (*n* 171), and one removing those that recorded a change in appetite in previous 2 weeks (*n* 834).

## Results

### Dietary data quality control

The mean code error rate detected through the initial 10 audit cycles was 3.7% (SD 3.2%) errors per food diary. The mean error range across 10 audit cycles was 2.8% (SD 3.5%) to 5.9% (SD 4.1%) with only one food diary exceeding the 10% code error limit (10.2% error rate). Analysis of audit cycle errors found that the most frequent coding errors were portion weight errors (55% of errors detected), followed by code selection errors (31% of errors detected). Common errors detected during the audit cycles shown in online supplementary material table S2. Gross coding errors were detected in 12% of food diaries coded.

### Population characteristics

The proportion of food diaries completed for the entire 7-day period was 88%; mean period of diary completion was 6.8 (SD 0.7) days. The characteristics of the sample by sex are shown in table 1. Men accounted for 63% of the sample and were significantly older than women: 42.4 (SD 8.9) vs 39.9 (SD 9.5) years, p<0.0001, and had a higher BMI: 27.7 (SD 3.6) kg/m^2^ vs 25.6 (SD 4.6) kg/m^2^, p<0.0001. Women reported a significantly lower mean daily EI compared with men: 1711 (SD 395) kcal per day versus2107 (SD 502) kcal per day; p<0.0001. There were significant differences between sources of EI between men and women, with women deriving more energy from carbohydrates (p<0.0001) and men deriving more energy from fats (p=0.004), proteins (p=0.001) and alcohol (p<0.0001).

**Table 1:** Characteristics of participants with dietary records from the Airwave Health Monitoring Study

	Men (*n* 2568)		Women (*n* 1510)		
	Mean	SD	Mean	SD	p Value
Age at screening, years	42.4	8.9	39.9	9.5	<0.0001*
Body mass index, kg/m^2^	27.7	3.6	25.6	4.6	<0.0001*
Basal metabolic rate, kcal per day	1906.0	159.0	1431.0	124.0	<0.0001*
Daily energy, kcal	2107.0	502.0	1711.0	395.0	<0.0001*
Energy intake: basal metabolic rate	1.1	0.3	1.2	0.6	<0.0001*
Macronutrient breakdown of energy intake	Median	IQR	Median	IQR	
% Energy intake carbohydrate	43.9	6.3	44.9	6.3	<0.0001*
% Energy intake protein	17.0	9.5	16.7	7.7	0.001*
% Energy intake total fat	33.6	5.3	31.4	5.5	0.004*
% Energy intake saturated fat	12.3	2.8	12.5	2.8	0.053*
% Energy intake alcohol	4.3	7.4	3.1	6.4	<0.0001†
% Classified under-reporting energy intake	56.0		41.0		<0.0001‡

*Student's t-test compared mean values between male and female participants.

†Mann-Whitney U test compared median values between male and female participants.

‡χ^2^ test compared differences between men and women for plausible and under-reporters of energy intake.

### Classification of under-reporting

Sex was significantly associated with under-reporting EI with 56% of men compared with 41% of women being classified as under-reporting EI (p<0.0001). The overall prevalence of likely under-reporting of EI was 49%. Sex-stratified analyses showed differences in the associations between demographic, lifestyle and occupational factors when under-reporters and plausible reporters were compared as presented in table 2. Across men and women, potential under-reporters were more likely to be classified as overweight or obese compared with plausible reporters (p<0.0001), and to be in the highest category for physical activity (p<0.0001). Male police staff were more likely to be classified as plausible reporters, while constable and sergeants were more likely to be classified as under-reporters (p=0.038). Men in the highest quartile for weekday sitting (10 to 13 hours per day) were more likely to be classified as a plausible reporter compared with the lowest quartile (<4 hours per day) (32% vs 21%, p<0.0001). For women, but not men, those classified as under-reporting EI were younger than plausible reporters: 39.2 (SD 9.4) vs 40.3 (SD 9.5) years, p=0.034. Coders with a higher mean error rate were more likely to code food diaries classified as under-reporting EI compared with those with a lower error rate (p=0.007). Ethnicity, household income, diary completion, education, smoking status, length of working week, shift work and job characteristics were not significantly associated with probable under-reporting for either men or women.

**Table 2:** Comparison of demographic, anthropometric, lifestyle and occupational characteristics of under-reporters and plausible reporters of energy intake across men and women in the Airwave Health Monitoring Study

	Men					Women				
	Plausible reporters		Under-reporters			Plausible reporters		Under-reporters		
	Mean	SD	Mean	SD	p Value*	Mean	SD	Mean	SD	p Value*
Age at screening, years	42.7	9.2	42.1	8.6	0.057	40.3	9.5	39.2	9.4	**0.034**
Basal metabolic rate (BMR), kcal day^-1^	1863	152	1940	157	**<0.0001**	1415	112	1454	138	**<0.0001**
Daily energy intake (EI), kcal	2497	430	1800	300	**<0.0001**	1942	314	1380	227	**<0.0001**
	n	%	n	%	p^∼^	n	%	n	%	p^∼^
Ethnicity (*n* 4075)					0.338					0.295
Caucasian	1072	95	1343	94		861	97	589	95	
Non-Caucasian	61	5	90	6		31	3	28	5	
Food diary completion					0.572					0.704
No, <7 days completed	132	11	157	12		100	11	73	12	
Yes, 7 days completed	1001	89	1278	88		793	89	544	88	
Body mass index										
Healthy (<25kg/m^2^)	342	30	217	15	**<0.0001**	508	57	277	45	**<0.0001**
Overweight (25—30kg/m^2^)	634	56	809	56		282	32	230	37	
Obese (>30kg/m^2^)	157	14	409	29		103	11	110	18	
Level of education (*n* 4077)					0.091					0.058
Left school before taking O levels	41	4	73	5		24	3	23	4	
GCSE/O-Level/CSE’ then education	329	29	448	31		244	27	188	30	
Vocational qualifications	82	7	101	7		60	7	54	9	
A levels / higher or equivalent	359	32	464	32		292	33	201	33	
Bachelor degree or equivalent	255	23	264	18		205	23	121	20	
Postgraduate qualifications	67	6	84	6		68	8	30	5	

	Men					Women				
	Plausible reporters		Under-reporters			Plausible reporters		Under-reporters		
	n	%	n	%	p Value †	n	%	n	%	p Value †
Physical activity level (METs category)					**<0.0001**					**<0.0001**
Low	183	16	95	7		164	18	51	8	
Moderate	544	48	598	42		462	52	282	46	
High	406	36	742	51		267	30	284	46	
Smoking status (*n* 4066)					0.891					0.071
Never	791	70	1004	70		630	71	413	67	
Former	262	23	324	23		188	21	131	21	
Current	76	7	102	7		74	8	71	12	
Annual household income (*n* 4077)					0.122					0.879
<£32 000	106	9	120	8		244	27	165	27	
£32 000—£47 999	140	12	152	11		93	10	59	10	
£48 000—£57 999	510	45	615	43		273	30	200	33	
£58 000—£77 999	265	23	388	27		182	20	130	21	
>£78 000	112	10	159	11		101	11	65	10	
Marital status (*n* 3944)					0.259					**0.002**
Cohabiting	154	14	218	15		150	18	151	25	
Divorced	36	3	53	4		78	9	35	6	
Married	811	72	1009	71		452	53	287	48	
Separated	37	3	30	2		20	2	17	3	
Single	80	7	108	8		161	18	106	18	
Shift work status (*n* 536)					0.222					0.121
No	57	34	78	42		66	60	35	49	
Yes	111	66	108	58		45	40	36	51	

	Men					Women				
	Plausible reporters		Under-reporters			Plausible reporters		Under-reporters		
	n	%	n	%	p Value †	n	%	n	%	p Value †
Police employment rank (*n* 3626)					**0.043**					0.760
Police staff	222	22	232	18		436	56	297	54	
Police constable/sergeant	685	67	904	70		317	41	237	43	
Inspector/chief inspector	93	9	126	10		23	3	15	3	
Superintendent or above	14	1	31	2		3	0	1	0	
Characteristics of job role (*n* 2875)					0.393					0.425
Mainly office-based duties	458	42	337	40		212	55	312	58	
Mainly mobile duties	635	58	506	60		171	45	226	42	
Working hours per week					0.129					0.120
<41 hours per week	420	37	504	35		586	66	381	62	
41 to 48 hour per week	444	39	539	38		215	24	150	24	
49 to 54 hours per week	138	12	187	13		47	5	38	6	
55 or more hours per week	129	11	205	14		45	5	48	8	
Quartile (Q) of hours sitting per weekday					**<0.0001**					0.638
Q1 <4 hours per day	234	21	324	23		210	24	155	25	
Q2 <4 <6 hours per day	295	26	420	29		254	28	160	26	
Q3 ≥6 <10 hours per day	243	21	353	24		229	26	154	25	
Q4 ≥10 ≤13 hours per day	361	32	338	24		200	23	148	24	
Coder mean error rate (*n* 4236)‡					**0.007**					0.146
Below average (<3.7% errors)	805	75	971	70		662	77	438	74	
Above average (≥3.7% errors)	267	25	413	30		193	23	153	26	

GCSE, General Certificate of Secondary Education; CSE, Certificate of Secondary Education; METs, Metabolic equivalents.

^*^Student's t-test compared mean values between continuous parametric variables.

†χ^2^ test compared the differences between categorical variables. Sample size =4078 unless otherwise stated, due to incomplete data collection for some variables.

‡not all coders were selected via the random quality check.

Stepwise logistic regression models showed that BMI, physical activity and age were significant predictors of EI under-reporting classification for men and women. Additional predictors for women were education and marital status, and weekday sitting for men. Across both sexes, BMI and physical activity were the variables that accounted for the highest increase in odds of being classified as an under-reporter. Those with a BMI of 30kg/m^2^ or more had higher odds of being classified as an under-reporter: OR 2.66 (95% CI 1.85 to 3.83) and 5.20 (95% CI 3.92 to 6.89) in women and men, respectively, compared with those with healthy BMI. Men and women in the highest physical activity category compared with the lowest were more likely to under-report: men OR 3.33 (95%CI 2.46 to 4.50); women OR 4.34 (95%CI 2.91 to 6.55) as shown in table 3. To explore if the association between coder error rate and under-reporting was subject to potential confounding, we conducted additional logistic regression models for men and women adjusted for the variables identified in the stepwise logistic regression models. After adjustment, we did not find that a higher coder error rate was associated with increased OR of dietary intake misreporting (data not presented).

**Table 3:** Predictors of under-reporting energy intake by sex*in the Airwave Health Monitoring Study

Women *(n 1286)*		
Predictor included in final model†	OR	95% CI
Body mass index		
Ref: healthy (<25 kg/m^2^)	1.00	
Overweight (25–30 kg/m^2^)	1.74	(1.34 to 2.27)
Obese (>30 kg/m^2^)	2.66	(1.85 to 3.83)
Physical activity/week		
Ref: low	1.00	
Moderate	2.06	(1.39 to 3.06)
High	4.34	(2.91 to 6.55)
Marital status		
Ref: cohabiting	1.00	
Divorced	0.45	(0.26 to 0.79)
Married	0.63	(0.46 to 0.87)
Separated	0.96	(0.45 to 2.06)
Single	0.69	(0.48 to 1.00)
Level of education		
Ref: left school before taking O levels	1.00	
GCSE/O-level/CSE	0.52	(0.25 to 1.10)
Vocational qualifications	0.61	(0.27 to 1.40)
A levels/higher or equivalent	0.40	(0.19 to 0.84)
Bachelor degree or equivalent	0.35	(0.16 to 0.75)
Postgraduate qualifications	0.25	(0.11 to 0.60)
Age (per 5-year increase)	0.91	(0.84 to 0.98)
Men *(n 2268)*		
Body mass index		
Ref: healthy (<25 kg/m^2^)	1.00	
Overweight (25—30 kg/m^2^)	2.38	(1.90 to 2.96)
Obese (>30 kg/m^2^)	5.20	(3.92 to 6.89)
Physical activity/week		
Ref: low	1.00	
Moderate	2.02	(1.50 to 2.73)
High	3.33	(2.46 to 4.50)
Quartile (Q) of hours sitting per weekday		
Ref: Q1 <4 hours per day	1.00	
Q2 <4 <6 hours per day	1.10	(0.86 to 1.41)
Q3 ≥6 <10 hours per day	1.08	(0.83 to 1.40)
Q4 ≥10 ≤13 hours per day	0.70	(0.55 to 0.90)
Age (per 5-year increase)	0.94	(0.89 to 0.99)

CSE, Certificate of Secondary Education; GCSE, General Certificate of Secondary Education; O-level, ordinary level.

*Logistic regression model analyses conducted for men and women separately.

†Variables included in model presented showed significant association (p <0.05) with under-reporting in stepwise logistic regression.

### Sensitivity analyses

Two sets of sensitivity analyses (data not shown) were conducted: (1) omitting participants reporting a special diet not for weight loss and (2) omitting participants reporting a change in appetite over the last 2 weeks. Neither of the analyses modified the prevalence of potential under-reporting EI. In models excluding participants who reported being on a special diet not for weight loss, ethnic category was a significant predictor of under-reporting for women, with British Caucasians compared with other ethnic categories more likely to be classified as under-reporting EI (OR 0.51 95% CI 0.27 to 0.97). In analyses removing participants reporting a change in appetite over the last 2 weeks, there was attenuation in regression estimate for weekday sitting in men, and in women, marital status was no longer a significant predictor of under-reporting. BMI and physical activity remained the strongest predictors of under-reporting in all sensitivity models.

## Discussion

A key strength of the Airwave Health Monitoring Study is the use of 7-day food diaries to measure dietary behaviour in this large single occupation cohort. Here we provide a detailed account of the standard operating procedure developed to code the Airwave Health Monitoring Study dietary data. Dietary assessment based on self-report is inherently limited by human error (participant recall or recording, reactivity to being under surveillance), coding errors (subjective decision-making) and the limitations of nutritional databases (increasing complexity of foods consumed). Therefore, understanding sources of potential error and bias and developing a robust dietary coding methodology is essential in the generation of reliable nutritional data. We also report the prevalence of likely under-reporting of EI and its associated characteristics in a cohort of British police force employees.

### Dietary data generation

To reduce intercoder error, due to the large number of coders required, and to improve coding reliability, we have developed a standard coding protocol, staff training and audit procedure. Additionally, to overcome the inherent limitations of using nutritional databases, namely that they can become outdated as food consumption becomes more varied, we have developed a standard codebook that we continually update as new foods are recorded. The mean EI and macronutrient sources of energy we have reported are comparable with those reported in the National Diet and Nutrition Survey (NDNS).^28^ The mean daily EI reported in the Airwave Health Monitoring study is 1711 (SD 395) and 2107 (SD 502) kcal for women and men, respectively, compared with 1560 (SD 442) and 2032 (SD 617) kcal for adults in the NDNS.^28^ Online supplementary material table S3.

Random errors may contribute to inaccurate intakes of energy and nutrients in the coding process; however, with the large sample size of the present study, we did not find coder error rate significantly associated with classification of under-reporting. The rigorous coder training, standard protocol and audit cycle have maintained a mean error rate below 10% per food diary checked. It is difficult to compare this value with other nutritional epidemiological studies as in-depth quality control results are rarely reported. Although actual error rates were not published, INTERMAP capped line errors at 6%; however, this was based on 24-hour recall data, which cannot be directly compared with 7-day estimated weight food diaries.^20^ For example, during 24-hour recalls, participants can be probed to clarify intake information, therefore providing a more detailed record for coding.

In the present study, we did not find any association between EI misreporting and the number of food diary days completed. It has been suggested that study participants may experience ‘experimenter effect’ at the start of the dietary recording (days 1 and 2) and diary ‘fatigue’ at the end of the study period (day 7).^29^ Exploratory analyses (data not shown) found lower EIs on day 7 compared with day 3 of the food diary records suggesting possible ‘diary fatigue’ which will require further investigation. However, we did not observe that partcipants who reported a lower intake at day 7 (compared with day 3) to be more likely to be classified as under-reporting EI.

### Prevalence and characteristics of EI misreporting

The gold standard method to measure energy expenditure is doubly labelled water; however, this method is expensive and not feasible in large-scale population studies. By using the Goldberg method, one of the most common statistical methods for classifying implausible dietary energy reporting, we estimate that the prevalence of potential EI under-reporting to be 49% in the Airwave Health Monitoring Study cohort. As there is no valid or consensus statistical method to measure energy under-reporting in large-scale surveys published, prevalence rates vary greatly making comparison between studies problematic. The prevalence we have reported is comparable with that reported in the general British population. Applying individualised PAL values and calculating 95%CI to estimate acceptability of EI reporting Murakami *et al*^30^ classified 45% men and 55% women as under-reporting EI from 7-day weighed food diaries. However, these rates are greater than those published in a review of EI misreporting that found the prevalence of under-reporting to be between 12 and 44% in studies using estimated food records conducted for 3, 4 or 7 days.^31^ Studies included in the review applied estimated PAL values of between 1.05 and 1.35. The PAL applied can be arbitrary in the absence of objective PAL measurement information; because of the way that the Goldberg equation derives the under-reporting cut-off points (assuming energy balance), the PAL used will impact the prevalence of under-reporting. Owing to the likely heterogeneous job roles within the Airwave Health Monitoring Study cohort (eg, office based, on the beat officers and mobile patrol), we decided to use the MET data to estimate PAL levels rather than apply a universal value. The higher prevalence of under-reporting that we have observed in comparison with some previous studies is likely due to the considerably higher PAL values (1.4, 1.6 and 1.8) we have applied to the Airwave Health Monitoring Study Cohort. A PAL of 1.4 was selected as the lowest value as it is representative of a man or a woman with a sedentary non-occupational activity level and light occupational level of activity Therefore, the positive association between higher PAL and possible under-reporting that we observed may be due to the self-reported measure of physical activity used.

A systematic review found that self-report physical activity measurements have low to moderate correlations with direct measurements with under-reporting and over-reporting of physical activity observed and no bias towards a specific population group.^32^ In the current study, we applied identical MET values for each activity level recorded to all classes of BMI based on IPAQ-SF guidelines (3.3 walking, 4.0 moderate-intensity activity and 8.0 vigorous-intensity activity). A recent study has suggested that calculating METs using the standard resting oxygen uptake of 3.5 mL O_2_^-1^ min^-1^ could overestimate energy expenditure in overweight and obese people by up to 38.8%,^33^ consequently overestimating under-reporting in these individuals. Systematic overestimation of METs in obese participants may result in misclassification of PAL category, subsequently overestimating and biasing classification of under-reporting in obese participants.

In agreement with previous studies, we observed that classification of under-reporting EI was biased towards specific population groups. Consistent with European Prospective Investigation into Cancer (EPIC)-Norfolk^34^ and UK NDNS,^9^ we found under-reporting prevalence to be directly associated with BMI. In agreement with a previous study conducted in a working-age French cohort, higher education in women, but not men, was associated with plausible EI reporting.^35^ We found advancing age to be a weak but significant predictor of plausible EI reporting, in agreement with findings from the EPIC cohort,^11^ although other studies have shown advancing age to be associated with under-reporting.^10^ The Whitehall II study reported that those in lower employment grades were more likely to be classified as reporting low EIs, following adjustment for BMI.^36^ However, in our logistic regression models, we did not find the occupational variables of rank, or job role to be significantly associated with classification of under-reporting EI after adjustment for established confounders. Higher mean weekday sitting hours for men was associated with reduced odds of being classified as under-reporting. Participants in the highest quartile for weekday siting were more likely to be in a job role that is predominantly office based potentially making it more practical for the participant to record dietary intake compared with being on mobile duties; however, in the current sample,∼30% of participants did not have these data available potentially explaining why job role was not found to be a significant predictor of EI reporting.

To avoid the potential excessive exclusion of participants based on EI misreporting, a previous study compared a simplistic measure of dietary reporting plausibility with the Goldberg method.^37^ They reported that arbitrary cut-off points set at <500 kcal/day for under-reporting and >3500 kcal/day for over-reporting classified 1% as misreporters compared with 31% using the Goldberg equation.^37^ Moreover, excluding misreporters based on the Goldberg method, compared with the simple cut-off method did not substantially alter the relationship between recorded dietary intakes with biomarkers of intake.^37^ In the present study, we excluded two participants that had a mean intake of <500kcal/day. There were 32 (0.7%) participants that reported a mean EI of >3500 kcal per day. Based on the Goldberg equation, these 32 participants were classified as reliable reporters of EI, potentially due to the majority of these participants reporting moderate and high PAL.

### Future work

Although our results reinforce the suggestion that potential under-reporting of EI is not a result of random error but a systematic bias, it is important to note that our results may reflect in part, the result of the analytical procedure used to classify EI misreporting.^38^ In particular, the association between PAL and BMI with under-reporting are potentially subject to statistical artefact. An important feature of equations that use estimated energy expenditure to determine plausibility of energy reporting is that they need to be reflective of the population to which they are applied. In the absence of inexpensive and convenient biomarkers to determine EI, there is a need to investigate appropriate algorithms to determine BMR and energy expenditure of representative populations.

The present study only considers EI misreporting; however, under-reporting may not be distributed equally across all types of foods but may be biased towards ‘unhealthy’ foods.^39^

A large pooling study of cohort studies found that BMI was a strong predictor of protein and EI under-reporting against established biomarkers (24-hour urinary nitrogen and doubly labelled water).^40^ However, in the absence of effective biomarkers to use in large cohort studies, bias in reporting at the food level in free-living populations cannot be estimated using statistical methods, or therefore adjusted for in analyses. The collection of biological samples (spot urine and blood) as part of the Airwave Health Monitoring Study will allow for future exploratory investigations into the use of biomarkers to aid the assessment of dietary intake in large epidemiological studies.

### Study limitations

There are a number of limitations specific to the current study. First, previous research has shown that those classified as restrained eaters are more likely to under-report their EI.^41^ A study investigating restrained eating in the UK NDNS reported that men in non-manual occupations were more likely to be classified as having a higher restrained eating score, and that in men, but not women, this trait was associated with under-reporting.^42^ Additionally, it has been suggested that stress may play a role in restrained eating behaviours.^43^ Therefore, as specific job roles within the police force may be associated with higher levels of stress, this could be an important consideration. However, we did not observe any difference in under-reporters, plausible reporters and job role. Although questions about dietary restraint^44^ were not included as part of the health screen, sensitivity analyses were conducted excluding those following a special diet not for weight loss or a change in appetite during the previous 2 weeks. These analyses did not change the overall prevalence of under-reporting of EI but did alter the significance of predictive characteristics. However, in both sets of sensitivity analysis, BMI and PAL remained the strongest predictors of under-reporting.

A further limitation of our current study is missing data on specific variables, in particular shift work that is only currently available for ∼12% of participants. However, shift work is highly associated with job role and rank within the cohort, neither of which were found to be a predictor of energy misreporting. Finally, it could be argued that the dietary data generated from the Airwave Health Monitoring Study cohort lacks external validity as it is based on a specific occupational group. However, with ∼250 000 people in Great Britain being employed by the police force in 2012,^45^ and with recent interest in reducing obesity in public sector workers,^46^ the data produced will give a valuable insight into dietary habits and potential measurement bias in comparable occupational groups such as paramedics and fire fighters.

## Conclusion

The Airwave Health Monitoring Study has collected the largest single occupation prospective dietary data set in the UK. Despite the acknowledged limitations of self-reported dietary intake, it is currently the only method of dietary measurement that is feasible for deployment in large-scale nutritional epidemiological studies. Here we provide a detailed account of the standard operating procedure developed to code the dietary data in the Airwave Health Monitoring Study, which we believe gives a useful insight into the practicalities of reducing coding error in large-scale dietary studies using food records. In agreement with previous studies, we observed the prevalence of under-reporting of EI to be directly associated with BMI. The reasons for this association are likely to be multifactorial and related to participant and methodological factors. However, with the potential for bias due to under-reporting it would be prudent to conduct sensitivity analyses^31^ or to adjust for EI 47 in analyses of dietary factors in relation to disease outcomes. The availability of blood and urine samples in the Airwave Health Monitoring Study, together with the dietary data, provides a valuable resource to investigate nutritional biomarkers for use in future studies.
