# Supplementary material for: Dietary assessment of British police force employees: a description of diet record coding procedures and cross-sectional evaluation of dietary energy intake reporting (The Airwave Health Monitoring Study)
Source: BMJ Open. 2017 Apr 4;7(4):e012927. doi: 10.1136/bmjopen-2016-012927 (PMC5388011; doi:10.1136/bmjopen-2016-012927)
Supplement: supplementary document [file bmjopen-2016-012927supp.pdf]

Airwave Health Monitoring Study

Standard protocol for food diary coding using Dietplan nutritional software

Developed by: Rachel Gibson, Rebeca Eriksen, Kathryn Lamb and Yvonne McMeel

Nutrition and Dietetics Research Group

Imperial College

London

Contents **Page**

**1. Introduction**	**3**
**2. Setting up the assessment on Dietplan**	**4**
**3. Selecting food codes**	**5**
**4. Portion size algorithms**	**15**
**5. Error checking**	**21**
**References**	**22**
**Appendices**	**23**

1.0 Introduction

The following pages provide an outline of how to input and code the food diaries from the Airwave Health Monitoring Study (example appendix).

The primary aim of this protocol is to reduce error and to standardise code and portion selection.

Please read through before you start to code and input the food diaries.

Other resources you will need to refer to for coding: Airwave Study unique food code book and the resources listed on page 22 of this document.

All coders should keep a weekly log of any issues regarding code / portion selection to discuss at the weekly meeting.

EXCLUSION CRITERIA for food diary entry into Dietplan:

- Less than one day completed
- Meal replacement diet recorded by the participant
- Incomplete diaries are coded but incomplete days are not e.g. if days 1-6 are complete but day 7 does not follow the same pattern as the previous days, days 1-6 are coded but 7 is not

If a diary is not entered onto Dietplan then please complete the Food Diary Log with the appropriate reason (Excel file)

2.0 Entering foods into Dietplan

Setting up day and meal structure

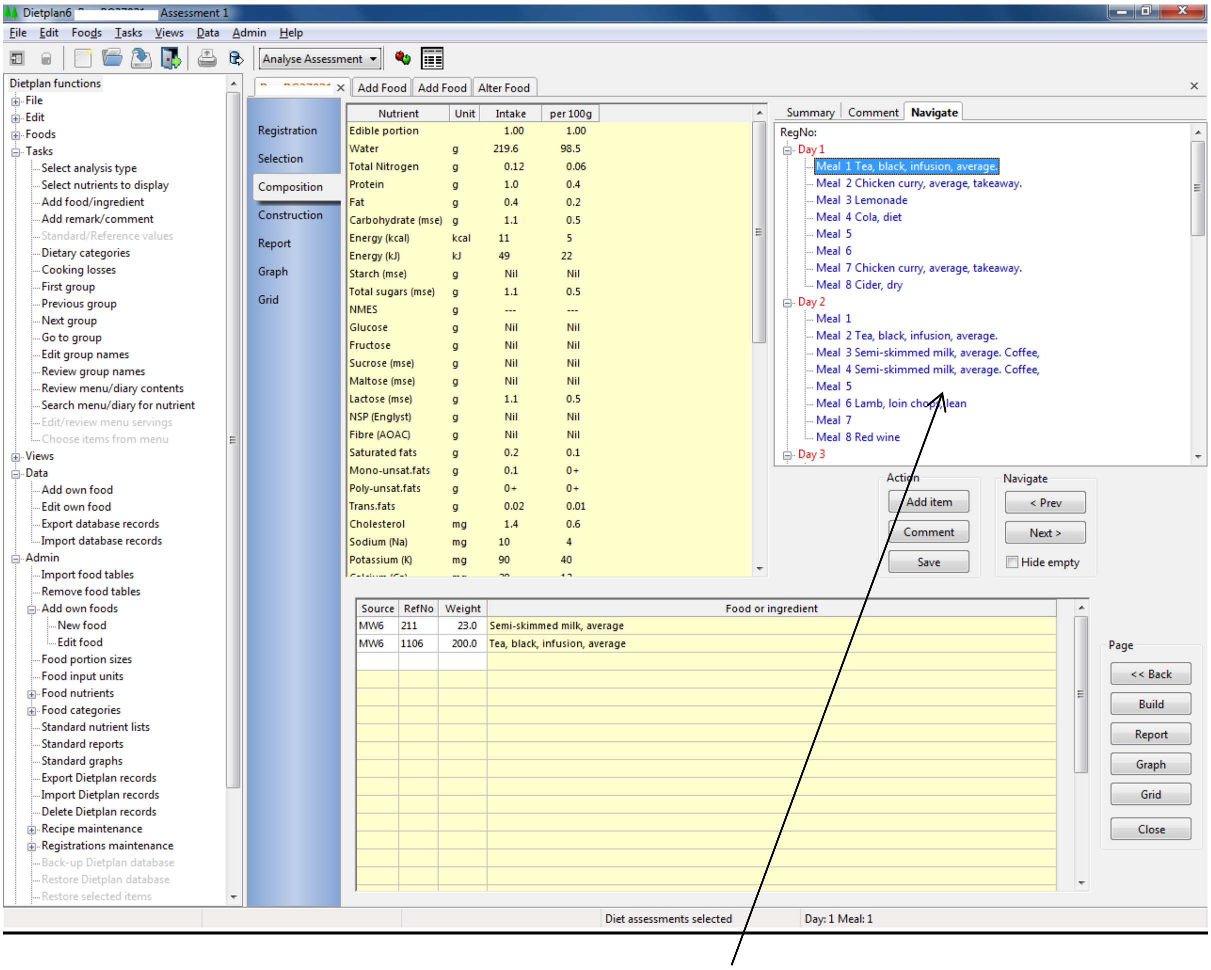

Enter each section of the daily diary under the meal numbers as follows:

Diet plan	Food diary section
Meal 1	Before breakfast
Meal 2	Breakfast
Meal 3	Mid-morning
Meal 4	Lunch
Meal 5	Tea
Meal 6	Evening meal
Meal 7	Later evening
Meal 8	'Anything else'.

Start from day 1 meal 1 and enter the items as written in the diary

- ✓ Remember to save your work as you go to avoid any data loss.

3.0 Selecting food codes

3.1 General rules

- ✓ UK Nutritional Dataset (UKN) is the default database. Only use (bespoke recipe) RCP and (user added foods, i.e. manual database entry (USF) databases if it's a food from the Airwave Health Monitoring Study (AW) codebook and prefixed with 'AW'
- ✓ Never select a code for an item that states "(fats only)" at the end of the description.
- ✓ Items such as drinks and sandwiches should be broken into their constituent parts
- ✓ Items should be coded in the form in which they were eaten e.g. raw tomato / boiled potato where possible unless the option is not available.

Algorithm for code selection:

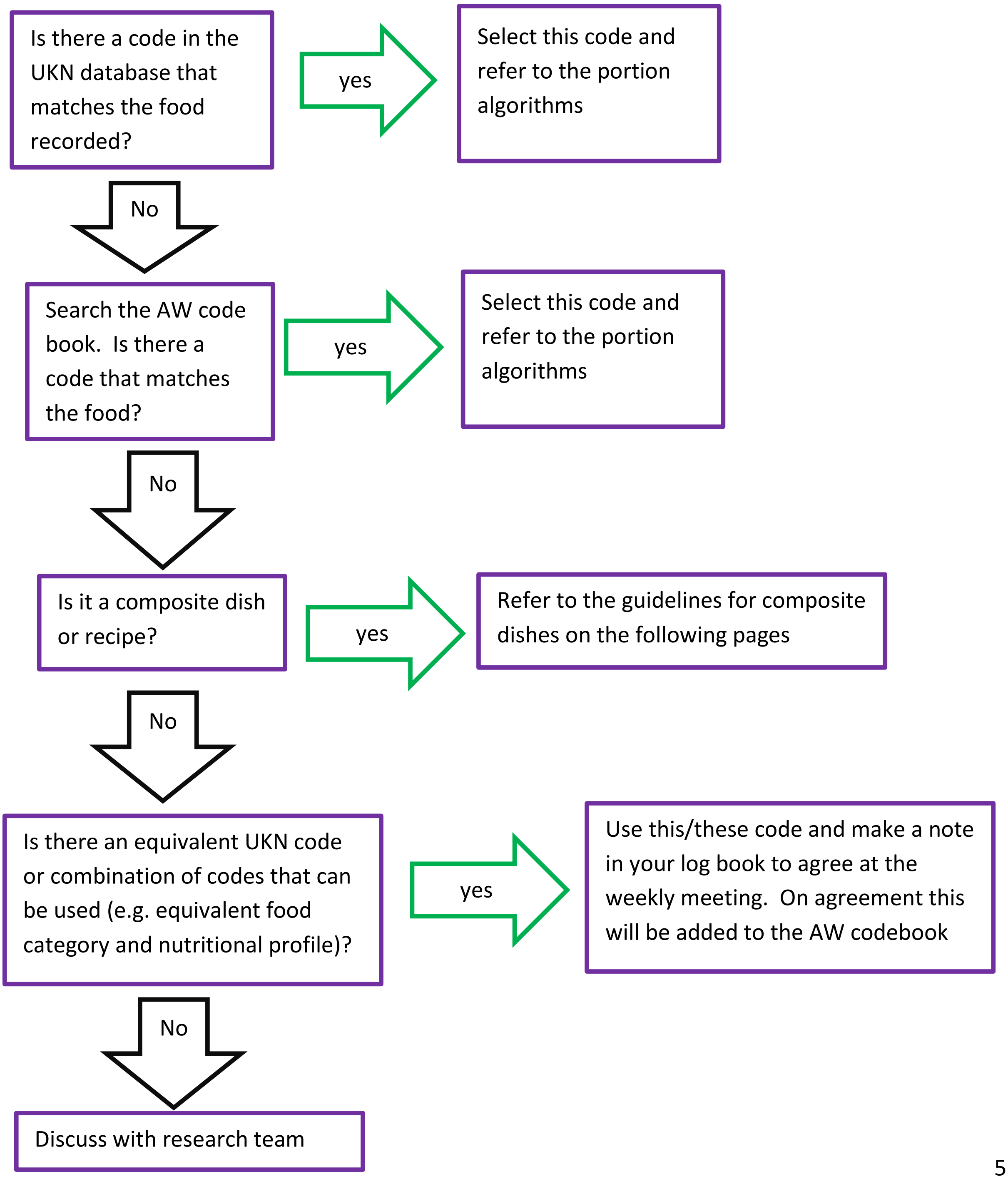

3.2 Composite Dishes

The following items should be broken into their constituent parts where possible:

- Hot beverages (except those made up with water)
- Sandwiches
- Home cooked meal when full recipe provided
- Homemade burgers

  - **Example 1:**

    Instant coffee with semi-skimmed milk and white sugar

    Enter as:

    UKN 17-159 instant, made up with water

    UKN 12-313 Semi-skimmed, pasteurised milk, average

    UKN 17-063 sugar, white

Rules for entering composite dishes:

1.Canned ready meals: i.e. spaghetti, soup, beans etc.

Select UKN code which states 'canned –reheated'.

2.Dish stated as 'homemade' and where a recipe is provided:

Enter all ingredients as the amount consumed and in their correct state– i.e. cooked / raw directly into the assessment (i.e. do not set up a separate recipe code).

3.Dish stated as 'homemade' and complex* recipe provided: i.e. ingredients are not available in their correct state in the database AND the recipe provided is not comparable to those in the UKN database. Check the standard recipes used if listed in McCance and Widdowsons (2).

*e.g. recipe with full quantities provided, but difficult to estimate actual proportion eaten.

In this instance a recipe should be set up using the 'Recipe analysis' option:

The code for the recipe should be the participant code prefixed by AW.

If multiple recipes are required for one participant then add 'a', 'b', 'c'… etc.. at the end of the code.

e.g. AW1234a

The recipe name should state the description of the dish and the participant code

e.g. Fruit cake – recipe for AW1234

Please ensure that the correct 'group code' is assigned to the recipe.

Please note these recipes should not be included in the Airwave codebook as they are unique to each participant.

4.Dish stated as ‘homemade ‘ and no recipe provided:

Select UKN code which states a ‘homemade’ version of this dish, if available.

5.Ready/Microwave meal (including ready to eat pizza, pies) with brand name provided:

Check the retailer / manufacturer website and select the closest equivalent code from diet plan that reflects the food eaten and the macronutrient profile.

NB: this may involve selecting 2 or more codes to combine to provide a similar food/nutrient profile.

See below for how to match foods not on the database.

6.Fast food burgers:

Enter as the complete food (e.g. Big Mac etc.)

If there is not a code in UKN database please check the excel Airwave code book. If there is no code in the code book then a recipe will be required (see recipe set up protocol).

7. Retail sandwiches:

Do not use sandwiches in Dietplan, match the sandwich to those online. Sandwiches can be found on Tesco and Waitrose websites. Use Waitrose for M&S and Waitrose sandwiches only (i.e. Premium/luxury type sandwiches) and Tesco’s for all other brands if brand not stated use Tesco as default.

Foods and meals not in the database

- Sandwiches
- Weight watchers/ reduced fat, calorie, sugar etc. meals
- Foods from cafés and fast foods e.g. costa, burger king, KFC etc.

How to code

- Find the nutritional information:
    1. Check Airwave codebook for item
    2. Check Portion Information folder
    3. Check retailers website
    4. Search online

- Calorie match (see screen shot below)
    1. Click ‘use nutrient exchanges’ button
    2. Choose kcal
    3. Type in the amount of calories you want to match in ‘exchange value’
    4. Click ‘view composition’
    5. If the protein, carbohydrates, sugars, fat are with 10% this amount can be used

- If the values for protein, carbohydrates, sugars, fat are more than 10% different (higher or lower) than that stated on the retailer/manufacturer declaration:
    - Check what nutrient is outside of the 10% range, e.g. protein is too low
    - Add foods using the **ingredients list** (only include foods if they are on the ingredient list)
        1. If more protein is required add more of the protein source
            a. If carbohydrates are required add more of the carbohydrate source
        - Be careful as some e.g. pasta, bread also contain protein
        - If sugar is needed add sugar.
            - There are different types of sugar. Some products have ingredients which are the same e.g. inverted sugar, white sugar and honey, glucose fructose syrup so only code white sugar. **Be mindful of adding sugar as it will increase carbohydrates.**
    - If fat is needed add oil or fat (from the source of fat in the product), check saturated fat.
        - One type of fat may not be suitable, both palm oil and rapeseed oil may be required.

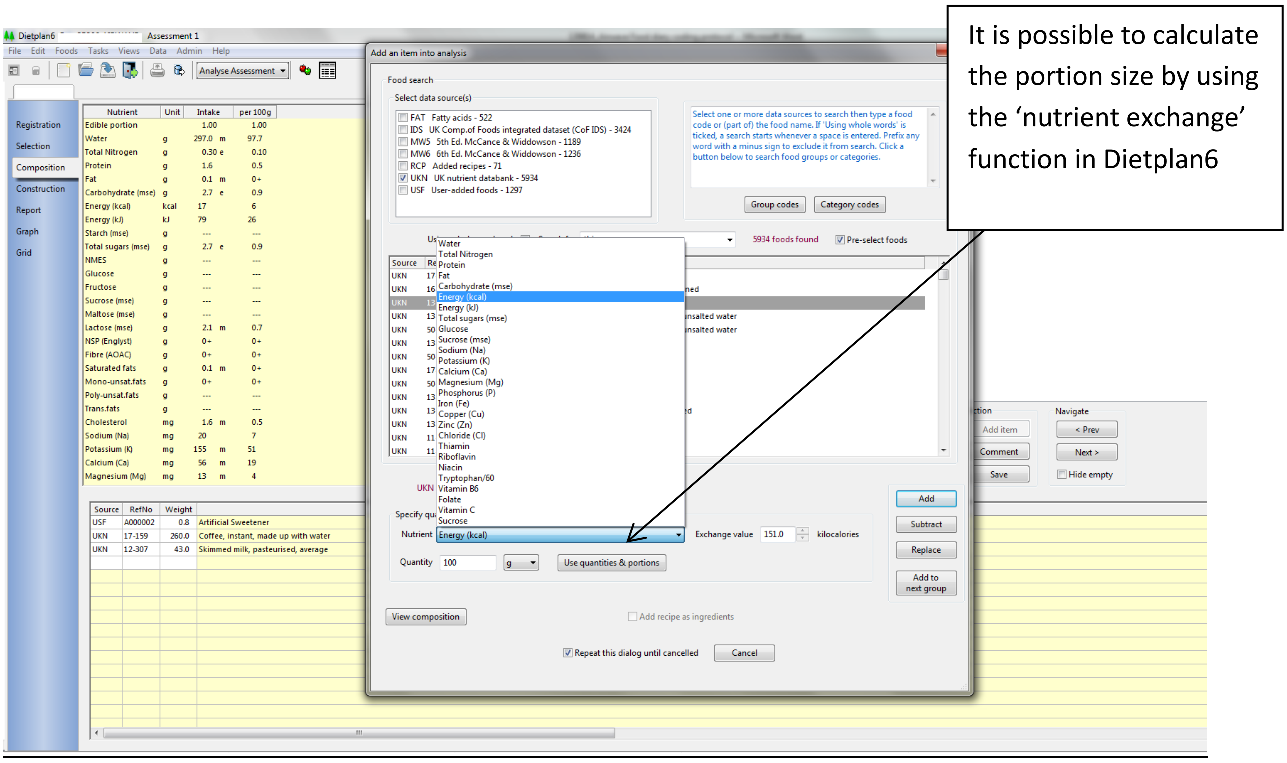

<u>Some foods e.g. Burger King burger's, pasta salads, Subway, sandwiches must be coded separately</u>

- Code the main ingredients using the ingredient list
    1. Calorie match the ingredients using their primary nutrient

        E.g. a ham salad sandwich
        - Calorie match bread to carbohydrates
        - Calorie match ham to protein
        - Add green salad portion as for homemade sandwich
    2. Compare fats, carbohydrates, sugar, protein for the meal to nutritional information
    3. Adjust weights of ingredients so nutrients are with 10%
    4. Some extra ingredients from the ingredients list may need to be to be added e.g. oil or sugar

8. Homemade sandwiches:

Unless otherwise stated:

2x medium slices of bread (as per the general questions response in the back of the food diary or default code)
Fat spread (if stated or stated in the general questions in the back of the food diary): note the quantity is used for each slice
Filling: unless otherwise stated use 'average' amounts for cheese / meats / fish / egg
Unless otherwise stated 20g lettuce / 34g tomato / 23g cucumber
'Salad' – default portion 20g of 'green salad' (UKN 15-380)other additions to sandwiches use ~15g.

9. Mixed Salads

If there is not enough information provided to be able to enter the weight of each vegetable included use 'green salad' UKN 15-380, see code book for default portion sizes.

3.3 Cooking methods:

Not all the different cooking options for each food type are available in the UKN database; therefore a comparable cooking method may need to be selected if the one recorded in the food diary is not available.

Vegetables

'steamed' or 'microwaved'	- enter as boiled 'unsalted water' if 'steamed' is not available
'stir fried'	- if fried option is not available enter as 'raw' (select type of oil as specified in the general questions as the 'average amount', refer to the codebook for the default code)
'stir fried' in cook in sauce	- enter as boiled 'unsalted water' if 'steamed' is not available
'roasted'	- select 'baked' or grilled if available + oil (select type of oil as specified in the general questions as the 'average amount', refer to the codebook for the default code)
'casseroled' or 'stewed'	- select 'boiled'

'boiled' = default cooking method unless vegetables are eaten as part of a salad

Meat / fish

'steamed'	- enter as 'casseroled' or 'stewed' if 'steamed' not available
'fried'	- select 'grilled' + oil if fried option is not available (select type of oil as specified in the general questions as the 'average amount', refer to the codebook for the default code)
'stir fried in cook in sauce'	- enter as 'casseroled' or 'stewed'
'roast'	- enter as 'grilled' if 'roast' option not available
'grilled'	- enter as 'roast' if 'grilled' option not available

Default cooking methods if not specified:

Potato, root vegetables, peas - 'boiled' unsalted water

Meat and fish – grilled

3.4 Weight changes on cooking

- If the raw weight of a food is given the cooked weight needs to be calculated (to account for water loss during cooking) and entered into Dietplan.
- If the weight is written in the ' amount' column we assume this is a cooked weight unless it is part of recipe

Weight Gain

- Foods which gain weight upon cooking (they expand when cooked)
  - E.g. pasta, rice, couscous, lentils

- Find the water gain in McCance and Widdowson's The Composition of Foods
- The number given is the percentage of water gained, this means the amount of water added. Therefore if it gives +144 water gain the total is x2.44

  - E.g. Dried pasta boiled +123%

    200g dry pasta, water gain 123%

    200 x 2.23

    = 446g boiled pasta

    Or (an alternative method)

    200g + 123%

  - E.g. Dried rice boiled +172%

    241g dry pasta, water gain 172%

    241 x 2.72

    = 655.52g boiled rice

    Or

    241 + 172%

If water and a water gain ingredients are given in the recipe e.g. rice in a risotto, pasta in a soup

- Calculate the water gain
- Take the water gain away from the amount of water in the recipe
- Add the water gain to the weight of the ingredient which gains water

E.g. Rice in a risotto

Respondent has eaten 1/4 of the recipe: *raw rice 500g, vegetables 500g and water 1000g*

Water gain rice +172%

500g x 1.72 = 860g

Amount of water in recipe

1000g – 860g gained by the rice = 140g

Amount of rice in recipe

Rice 500g + water gained 860g

= 1360g

Respondent has eaten a quarter of: *boiled rice 1360g, vegetables 500g and water 140g*

Water loss

- Foods which lose weight upon cooking
    - E.g. meat, fish

- Find the water loss in McCance and Widdowson's The Composition of Foods
- The number given is the % of water lost during cooking
    - E.g. Raw stewing beef -36%

450g stewing beef, water loss -36%

450g x 0.36 = 162g lost

= 450g raw stewing beef - 162g loss

= 288g eaten

Or if using a calculator 450 – 36% = 288g

- E.g. Raw chicken casseroled

175g stewing beef, water loss -25%

175g x 0.25 = 43.75g lost

= 175g raw chicken – 43.75g loss

= 131.25g eaten

Or if using a calculator 175g – 25% = 131.25g

All sausages to be entered as cooked weights unless specified otherwise e.g. brand and weight. Use the following weights and NOT those stated in the UKN dietplan6 dataset.

Default sausage weights (each):

	raw	cooked
Thick	47g	35g
Cocktail	12g	9g
Thin	25g	19g
Premium (e.g.' best' / 'finest' / taste the difference'	65g	49g

(Based on average supermarket weight per sausage recorded in Foodbase (1) appendix 6)

Default cooked weight is not stated: 34g (average thick, thin and premium)

Conversion of liquids to grams:

- Liquids have a specific gravity so 100ml is not always 100g
- Conversion factors must be used as weight must be coded
- Used for milk, cream, ice cream, egg, oils, fizzy drinks, 100% fruit juices, alcoholic drinks
- Do not use for water, squash, tea, coffee

How to calculate the conversion factor

- All conversion factors are after the contents page in the FSA Food Portion Sizes book (from page viii)(3)
- Multiple value in 'specific gravity' column by the ml given

E.g. 200ml of whole milk

Conversion factor 1.031

200ml x 1.031

= 206.2g milk

E.g. 330ml can cola

Conversion factor 1.04

330ml x 1.04

= 343.2g cola

Tips

- If the respondent has used a small amount e.g. 3tbs of milk the conversion factor does not need to be used
- The weights of milk for tea and coffee in section 1.2 **do not** need a conversion factor
- If the amount has been given in ounces, litres or pints convert to ml first then use conversion factor (values are at the top of the same page as the conversion factors)
- Weights of glasses and cups are given in ml in this document
- Some alcoholic drinks have pints and half pints in the drop down, these already have the conversion factor so it does not need to be calculated again
- Be careful of yogurt as the respondent sometimes puts 125ml or 500ml, they probably mean 1 pot which is 125g or 500g so the conversion factor does not need to be used
- If a volume of food is given check the food density in Food and Agriculture (FAO) Foods Density Database Version (4).

Spoon volumes not listed in the Food Standards Agency (FSA) food portion book (3):

Serving spoon = 40ml. Ministry of Agriculture, Fisheries and Food (MAFF) Food Atlas (5)

Ladle = 120ml: mean volume of the two serving spoons featured in MAFF Food Atlas (5)

If a participant enters 'spoon' but does not specify what size, then apply appropriate default sizes:

Meat in sauce / vegetables – tablespoons

Vinegar / herbs / spices / sugar - teaspoons

Cup: for tea / coffee and other hot drinks refer to portion algorithm.

If a ‘cup’ is used to measure liquids other than hot drinks take the default volume to be 250ml (UK cooks measure) and apply specific gravities as appropriate.

Other arbitrary measures:

‘a handful’ of Fruit and vegetables code 100g

‘a handful’ of nuts and dried fruits code 30g

‘a splash of oil’ code 5g

‘a drizzle of salad dressing’ = 10g (25th percentile for salad dressing see reference Foodbase (1)

Imperial measures: Conversion of imperial measures to grams are provided in the FSA Food Portion Sizes book (3), page viii.

Note that 1cl = 10ml, so they might say 75cl of wine which is 750ml which is a standard bottle.

4.0 Portion size algorithms

Algorithm for portion size estimation – hot drinks

Has the respondent referred to a cup / mug?

yes →

Cup: for tea / coffee and other hot drinks = 190ml volume and apply specific gravities as required. (NB 190ml = tea / coffee without milk)

Mug: for tea / coffee and other hot drinks = 260ml volume and apply specific gravities as required. (NB 260ml = tea / coffee without milk)

Note: added milk use average amount 30g for tea and 43g for coffee

No ↓

Default to mug 260ml

NB:

Adding milk to hot drinks

Tea / coffee entered as the cup / mug volume (i.e. 190 / 260ml) then add the milk.

e.g. mug of tea with average milk

Tea infusion average 260g + semi skimmed milk pasteurised average 30g

Sugar in drinks

If a participant records '1 sugar' – enter 1 level teaspoon (=4g sugar).

Algorithm for portion size estimation – cold / ambient / alcoholic drinks described by a glass.

Has the respondent referred to a specific volume for example ½ pint?

yes → Use this volume and apply specific gravities as required.

No ↓

Has the respondent referred to small / medium or large glass?

yes →

Glass: If there is not a volume / weight in the FSA portion book (3) (pages 3-6) for the specified liquid use the following default volumes and apply specific gravities as required.

Non-alcoholic:

Large = 300ml (FSA book (3) volume for 'tumbler')

Average, medium or unspecified = 200ml volume (MAFF straight glass volume) (5)

Small = 160ml (MAFF small straight glass volume) (5)

Alcoholic:

Wine: small / medium 125ml (Foodbase 25/50th centile). Large 250ml - *Carbs and Cals* (6)

Beer / lager / cider: small 284ml / large 568ml

Spirits: refer to FSA food portion book (3) page 3 – 6.

A 'large' spirit is to taken as a double measure

No ↓

Use default volumes shown below and apply specific gravities as required.

200ml (non-alcoholic)

125ml (wine)

Beer / lager / cider: 430ml (average of pint + ½ pint).

<u>Algorithm for portion size estimation – cold / ambient / alcoholic drinks described by a bottle or can.</u>

Has the respondent referred a specific brand / tin size? Eg. Half a can of red bull?

yes → Use this volume and apply specific gravities as required.

No ↓

Has the respondent referred to small / medium or large bottle or can?

yes → Is there a volume / weight in the FSA portion book (pages 3-6) for the item.

- yes ↑ Use this volume and apply specific gravities as required.
- No ↓

<u>Non-alcoholic - based on FSA portion sizes(3):</u>

Can = 330ml

Small carton = 200ml

Large bottle = 1ltr

Medium bottle = 500ml

Small bottle = 250ml

<u>Alcoholic:</u>

Wine – small bottle = 375ml (FSA ½ bottle). Large bottle = 750ml

Beer / lager / cider: (based on common sizes listed at Sainsbury's / Tesco 02/08/13):

Small bottle = 275ml

Medium / regular bottle = 330ml

Large bottle = 1lt

Small can = 330ml

Medium can = 390ml

No ↓

Use default volume of average and apply specific gravities as required.

330ml can (non-alcoholic)

390ml can (lager, cider, bitter) – mean average of small and large can.

330ml bottle (beer / cider / lager)

<u>Algorithm for portion size estimation – food: canned and tinned, when recorded as a proportion of a can /tin.</u>

Has respondent referred a specific brand / tin size? E.g. Half a Heinz 400g tin of beans?

yes

Refer to the manufacturer or retailer website (e.g. Sainsbury's, Tesco) to find the appropriate tin weight.

No

Select from default tin size*: Non-drained weights.

<u>Fish:</u>

Tuna - 160g

Salmon - 105g

Sardine – 120g

<u>Vegetables / pulses:</u>

Tomatoes – 400g

Sweet corn kernels – 165g

Peas (not mushy) – 300g

Mushy peas – 300g

Beans (not baked) – 400g

Baked beans – 415g

<u>Other:</u>

Soup – 400g

Spaghetti in sauce – 400g

Fruit – 415g

*based on most common tin size featured on

Sainsbury's & Tesco website 02/08/13

Enter the '<u>drained weight' /'edible portion'</u> of the portion of the can eaten.

If you would normally consume the medium in which the food is canned the drained weight would = 100%.

e.g. baked beans, tinned tomatoes, soup, mushy peas, items tinned in tomato sauce and tinned fruit would = 100%.

If you would not normally eat the medium in which the food is canned (e.g. oil and brine) you need to calculate the drained weight:

Tuna / sardines / salmon canned in oil or brine = 82%

Sweet corn canned in water / salted water = 82%

Chick peas / tinned beans canned in water / salt water = 60%

Example calculation:

Half a 400g can of chick peas

= 400g/2 = 200g

= 200g x 0.60 = <u>120g</u> (weight to enter)

Algorithm for portion size estimation FOOD (NOT canned / tinned):

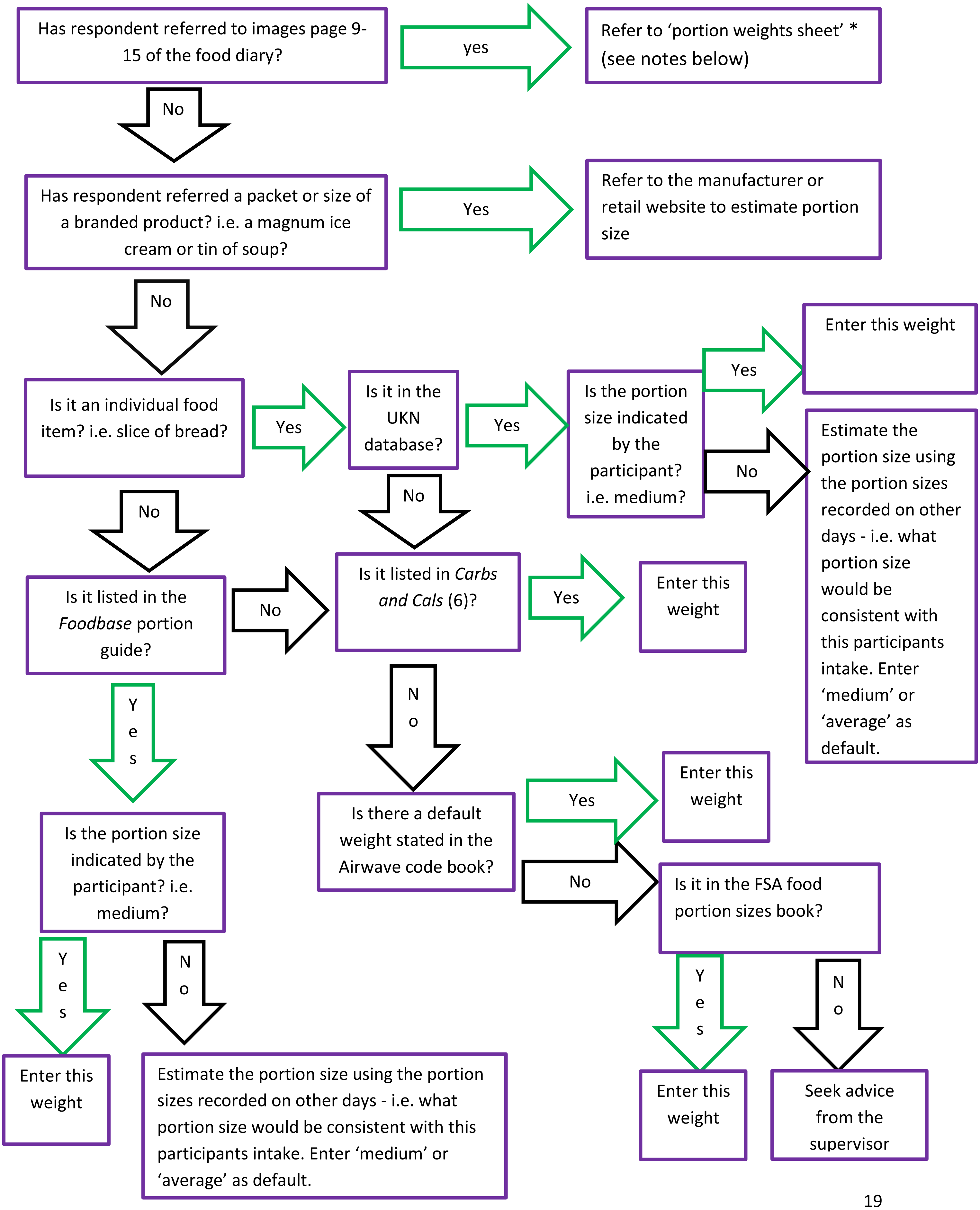

*Notes to the Airwave food portions

- Check that you select the portion size relative to the food type. For example picture set 14 related to salad and boiled leaf vegetables,

    e.g. photo 14a = 51g for boiled spinach

    = 15g for lettuce

- If a participant has only referred to one portion size against 2 foods that are from different food groups:

Eg. Chicken in curry sauce & boiled rice   8b

Select portion 8b for the rice (177g) and select the 'b' size portion for the chicken curry, in this example it would be 19b (115g).

- If a participant has only referred to one portion size against 2 foods that are from the same food group:

EG. Carrots, broccoli, green beans   13c

Select 13c = 107g divide by 3 = 36g per type of vegetable

5.0 Error checking

- Save the assessment
- Check the following against the diary you have inputted:
  - ✓ Registration code is the same as the code on the front of the diary you have inputted
  - ✓ Meal occasions have be entered in the correct meal number (1-8)
  - ✓ Codes represent the foods in the diary
  - ✓ Portion sizes are entered correctly
  - ✓ Any changes are saved

  ➔ Select ‘grid’

When grid view opens you can double check foods and quantities:

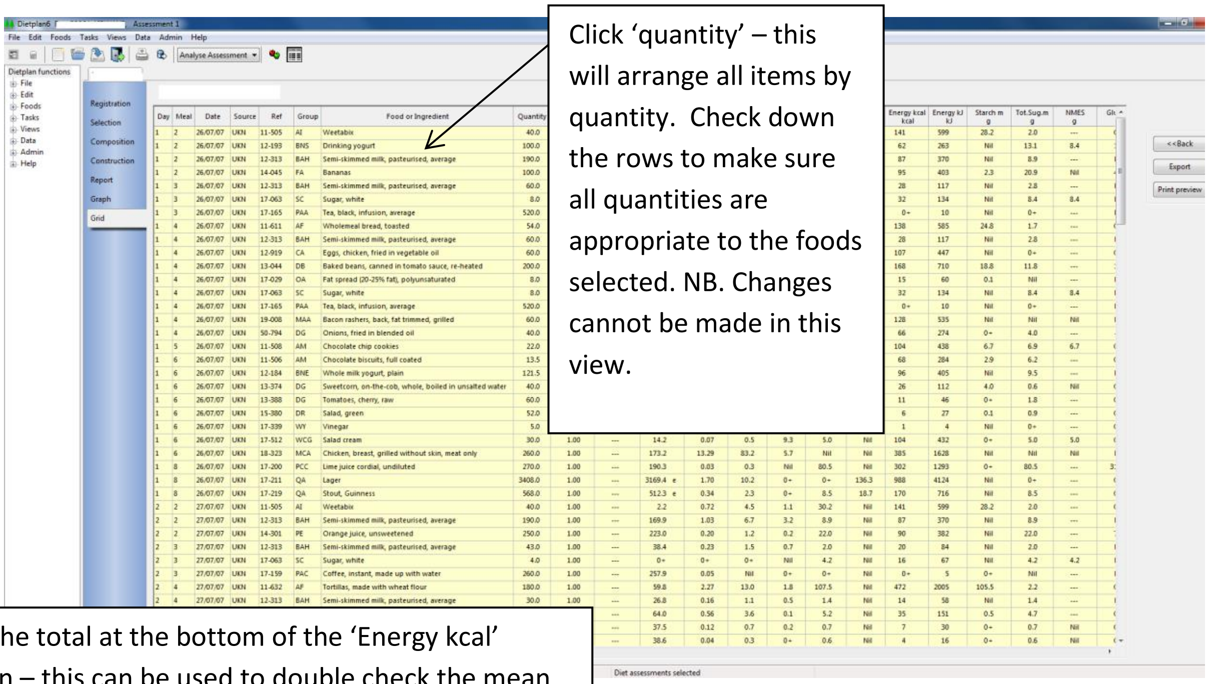

Click ‘quantity’ – this will arrange all items by quantity. Check down the rows to make sure all quantities are appropriate to the foods selected. NB. Changes cannot be made in this view.

Note the total at the bottom of the ‘Energy kcal’ column – this can be used to double check the mean kcal intake per day. Range should be approx. 1500 – 3000kcal

References:

1. Food Standards Agency. Foodbase 2005 [30.08.13]. Available from: http://www.foodbase.org.uk/results.php?f_report_id=82.
2. Food Standards Agency. McCance and Widdowson's: The composition of foods. 6th Summary ed. Cambridge: Royal Society of Chemistry; 2002
3. Food Standards Agency. Food Portion Sizes. 3rd ed. London: TSO; 2010.
4. Charrondiere R, Haytowitz D, Stadlmayr B. FAO/ FOODS Density Database Version 2.0. FAO; Rome, Italy, 2012
5. Nelson M, Atkinson M, Meyer J: *A Photographic Atlas of Food Portion Sizes*. London: MAFF Publications; 1997
6. Cheyette C, Balolia Y. Carbs and cals and protein and fats 1st ed. United Kingdom: Chello Publishing; 2010.

Acknowledgements:

Dietplan6.7 nutritional software programme (*Forestfield Software Ltd, Horsham, UK*)

Appendix

<u>Example pages from the Airwave Health Monitoring Study food diary record (front cover, example record, sample of portion size photos included, general questions included in the diary and used to guide coding – refer to separate document –‘ *Using the answers recorded in page 45-50 of the food diary to aid code selection’*)</u>

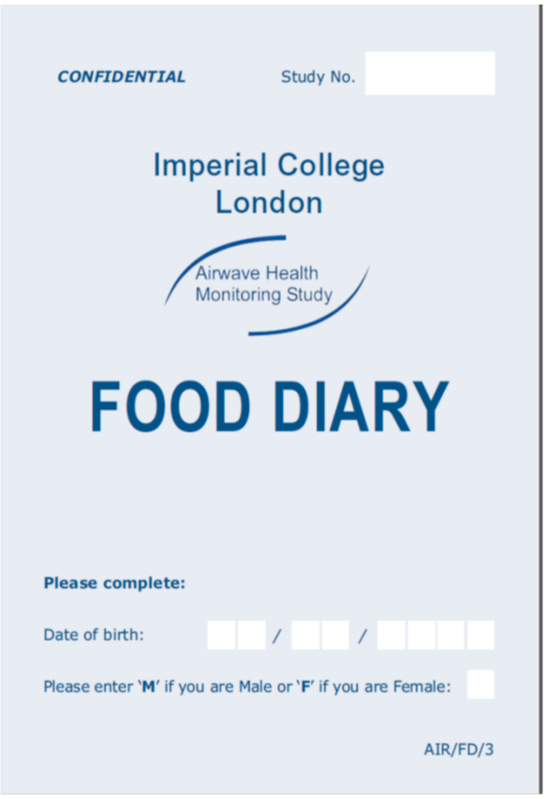

CONFIDENTIAL Study No.

Imperial College London

Airwave Health Monitoring Study

FOOD DIARY

Please complete:

Date of birth: / /

Please enter ‘**M**’ if you are Male or ‘**F**’ if you are Female:

AIR/FD/3

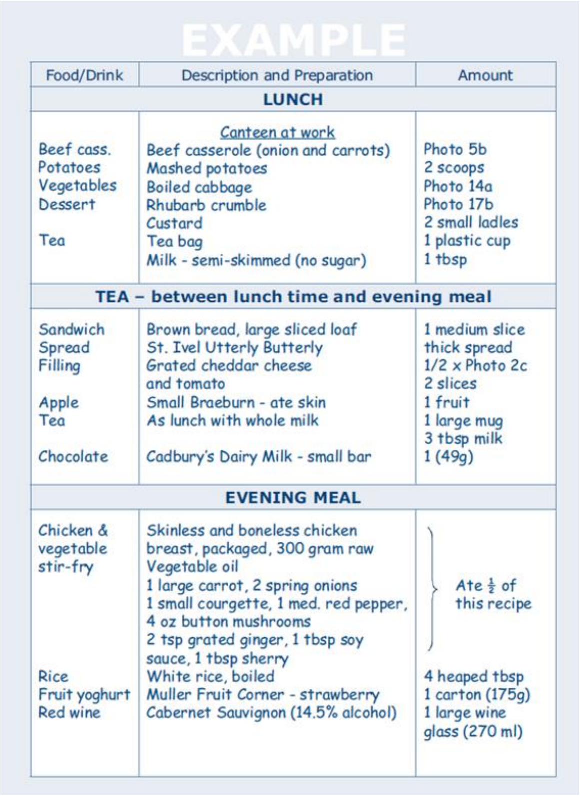

EXAMPLE

Food/Drink	Description and Preparation	Amount
	**LUNCH**	
	<u>Canteen at work</u>	
Beef cass.	Beef casserole (onion and carrots)	Photo 5b
Potatoes	Mashed potatoes	2 scoops
Vegetables	Boiled cabbage	Photo 14a
Dessert	Rhubarb crumble	Photo 17b
	Custard	2 small ladles
Tea	Tea bag	1 plastic cup
	Milk - semi-skimmed (no sugar)	1 tbsp
	**TEA – between lunch time and evening meal**	
Sandwich	Brown bread, large sliced loaf	1 medium slice
Spread	St. Ivel Utterly Butterly	thick spread
Filling	Grated cheddar cheese	1/2 x Photo 2c
	and tomato	2 slices
Apple	Small Braeburn - ate skin	1 fruit
Tea	As lunch with whole milk	1 large mug
		3 tbsp milk
Chocolate	Cadbury's Dairy Milk - small bar	1 (49g)
	**EVENING MEAL**	
Chicken & vegetable stir-fry	Skinless and boneless chicken breast, packaged, 300 gram raw<br>Vegetable oil<br>1 large carrot, 2 spring onions<br>1 small courgette, 1 med. red pepper,<br>4 oz button mushrooms<br>2 tsp grated ginger, 1 tbsp soy sauce, 1 tbsp sherry	Ate ½ of this recipe
Rice	White rice, boiled	4 heaped tbsp
Fruit yoghurt	Muller Fruit Corner - strawberry	1 carton (175g)
Red wine	Cabernet Sauvignon (14.5% alcohol)	1 large wine glass (270 ml)

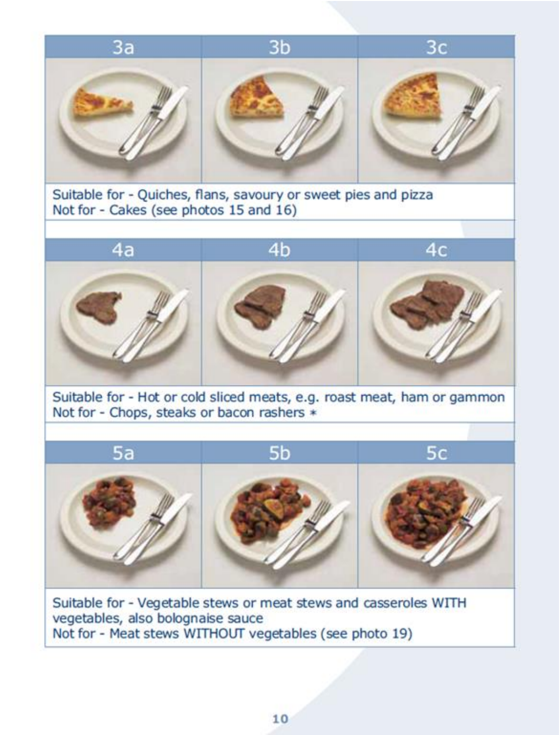

3a 3b 3c

Suitable for - Quiches, flans, savoury or sweet pies and pizza
Not for - Cakes (see photos 15 and 16)

4a 4b 4c

Suitable for - Hot or cold sliced meats, e.g. roast meat, ham or gammon
Not for - Chops, steaks or bacon rashers *

5a 5b 5c

Suitable for - Vegetable stews or meat stews and casseroles WITH vegetables, also bolognaise sauce
Not for - Meat stews WITHOUT vegetables (see photo 19)

10

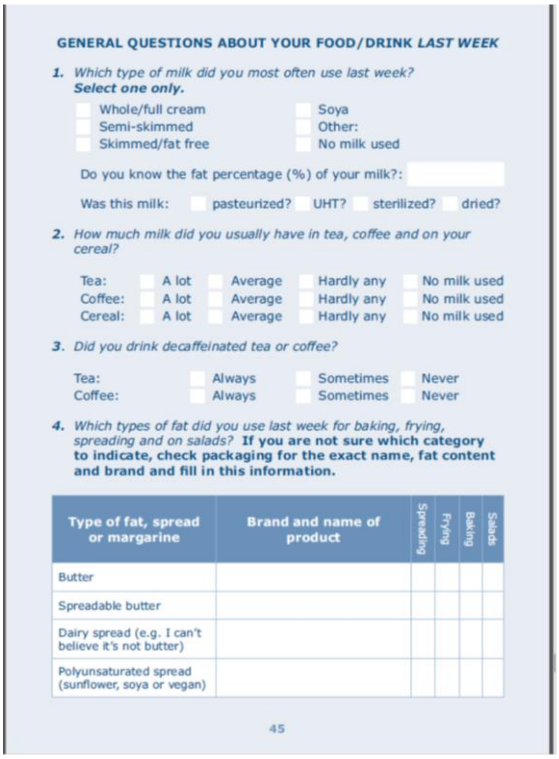

GENERAL QUESTIONS ABOUT YOUR FOOD/DRINK *LAST WEEK*

1. *Which type of milk did you most often use last week?* ***Select one only.***
   - Whole/full cream
   - Semi-skimmed
   - Skimmed/fat free
   - Soya
   - Other:
   - No milk used

   Do you know the fat percentage (%) of your milk?:

   Was this milk: pasteurized? UHT? sterilized? dried?

2. *How much milk did you usually have in tea, coffee and on your cereal?*

Tea:	A lot	Average	Hardly any	No milk used
Coffee:	A lot	Average	Hardly any	No milk used
Cereal:	A lot	Average	Hardly any	No milk used

3. *Did you drink decaffeinated tea or coffee?*

Tea:	Always	Sometimes	Never
Coffee:	Always	Sometimes	Never

4. *Which types of fat did you use last week for baking, frying, spreading and on salads?* **If you are not sure which category to indicate, check packaging for the exact name, fat content and brand and fill in this information.**

Type of fat, spread or margarine	Brand and name of product	Spreading	Frying	Baking	Salads
Butter					
Spreadable butter					
Dairy spread (e.g. I can't believe it's not butter)					
Polyunsaturated spread (sunflower, soya or vegan)					

45
