# Supplementary material for: Dietary assessment of British police force employees: a description of diet record coding procedures and cross-sectional evaluation of dietary energy intake reporting (The Airwave Health Monitoring Study)
Source: BMJ Open. 2017 Apr 4;7(4):e012927. doi: 10.1136/bmjopen-2016-012927 (PMC5388011; doi:10.1136/bmjopen-2016-012927)
Supplement: supplementary table [file bmjopen-2016-012927supp_table.pdf]

Supplementary Material

Table S1 Example scenarios and possible coding solutions when exact code matches between recorded foods and UKN* database codes are not available.

**Example food item**	**Scenario**	**Codebook solution**
White barm cake	Regional food name for a bread roll	UKN code for soft white roll
Hash brown	No exact name match. UKN database contains a different name	UKN code for potato cake
Reduced salt tomato sauce	Exact food item not available. Regular tomato sauce available.	Set up a new database code by adapting the UKN code for tomato sauce so that the sodium level reflects the manufacturer declaration for the low salt version.
Choc chip wheat biscuit breakfast cereal	Exact food item not available. Codes for constituent items available.	Use the UKN rule for wheat biscuit breakfast cereal (92% of estimated portion weight) and the UKN code for chocolate (8% of estimated portion weight).
Protein shake	No exact or similar food items available in the UKN.	Set up a new food item using the manufacturer nutrient declaration

*UK Nutritional Dataset based on McCance and Widdowson's 6th Edition Composition of Foods

Table S2 Examples of different errors found during quality checking procedures

**Category of coding error**	
**Weight / portion error**	**Code / item selection error**
Not following SOP for weights	Wrongly matched food code
Not applying correct weight loss/gain from cooking	Recipe provided in the diary not used
Not applying specific gravity to liquids	Incorrect type, milk, spread or bread coded (e.g. not using the information provided in the general questions section)
Spread not added to appropriate number of slices of bread	

Abbreviations: SOP standard operating procedure for food diary coding; UKN United Kingdom Nutritional food database.

Table S3 Comparison of mean dietary intakes recorded in Airwave Health Monitoring study sub cohort and the reported intakes from the National Diet and Nutrition Survey[1]

	**Sub cohort Airwave Health Monitoring Study**				**National Diet and Nutrition Survey[1]**			
	**Women**		**Men**		**Women**		**Men**	
**Daily energy, kcal, mean (SD)**	1711	395	2107	502	1560	442	2032	617
**Macronutrient breakdown of energy intake***								
% Energy intake carbohydrate, mean (SD)	44.9	6.4	43.9	6.3	46.3	7.9	44.0	7.6
% Energy intake protein, mean (SD)	16.7	3.0	17.0	3.3	16.5	2.5	16.5	4.8
% Energy intake total fat, mean (SD)	34.1	5.5	33.6	5.3	32.9	6.8	33.0	7.1
% Energy intake saturated fat, mean (SD)	12.5	2.9	12.3	2.8	12.1	3.5	12.1	3.6
% Energy intake alcohol, median (IQR) †	3.1	6.4	4.3	7.4	1.5	-	3.3	-
**Intake by nutrients**								
NSP (g/day)	12.1	4.1	14.1	5.1	12.8	3.9	14.9	5.6

IQR, Interquartile range; NSP, non starch polysacharides

(1) Bates B, Lennox A, Prentice A, *et al.* (2014) National Diet and Nutrition Survey: Results from Years 1-4 (combined) of the Rolling Programme (2008/2009 – 2011/12). London: TSO.

*Data shown for % of total energy intake

†Includes non consumers

21
